# Long-Term Survival after Choroidal Metastasis Following Colorectal Cancer Surgery: A Case Report

**DOI:** 10.70352/scrj.cr.25-0317

**Published:** 2025-07-02

**Authors:** Keiichiro Ryujin, Tetsuro Kawazoe, Shota Sato, Akihiko Otake, Yuki Shin, Hirotada Tajiri, Yoko Zaitsu, Koji Ando, Eiji Oki, Tomoharu Yoshizumi

**Affiliations:** Department of Surgery and Science, Graduate School of Medical Sciences, Kyushu University, Fukuoka, Fukuoka, Japan

**Keywords:** colorectal cancer, ocular metastasis, choroidal metastasis

## Abstract

**INTRODUCTION:**

Choroidal metastasis from colorectal cancer is extremely rare, accounting for approximately only 4% of all choroidal metastases. However, with the increasing incidence and improved survival rates of colorectal cancer, the importance of diagnosing and treating ocular metastases is growing. We report a case of choroidal metastasis from colorectal cancer and review the relevant literature.

**CASE PRESENTATION:**

A 67-year-old man underwent curative surgery and adjuvant chemotherapy for ascending colon cancer. Two years later, pulmonary recurrence was detected and surgically resected. At 2 years and 5 months postoperatively, he developed visual impairment in the left eye, which led to the diagnosis of choroidal metastasis. A combination of systemic chemotherapy and local radiotherapy resulted in tumor shrinkage and relief of ocular pain. With additional local treatments administered in response to subsequent recurrences, the patient achieved long-term survival—5 years and 6 months after surgery and 3 years after the diagnosis of choroidal metastasis. A review of 22 reported cases of choroidal metastasis from colorectal cancer published since 2000 revealed that most patients had multi-organ metastases at the time of diagnosis. The average survival following the diagnosis of ocular metastasis was 10.4 months, indicating a poor prognosis. By contrast, local treatments—such as radiotherapy and intravitreal injections—contributed to symptom relief and the maintenance of quality of life. This case represents a valuable example of long-term survival achieved through combined local therapies.

**CONCLUSIONS:**

Although choroidal metastasis from colorectal cancer is rare, clinical management should consider the possibility of ocular involvement. A multidisciplinary approach combining systemic therapy with local treatments is essential for maintaining quality of life and prolonging survival.

## Abbreviations

CME
: complete mesocolic excision
CTCAE
: Common Terminology Criteria for Adverse Events
MSS
: microsatellite stable
UICC
: Union for International Cancer Control

## INTRODUCTION

Colorectal cancer is the second leading cause of cancer-related mortality in developed countries, accounting for approximately 9% of all cancer deaths.^1)^ In Japan, it ranks first in cancer incidence (approximately 150000 new cases annually) and second in cancer-related deaths (approximately 50000 per year), with both figures exhibiting a continuous upward trend.^2)^

In cases without distant metastasis, colorectal cancer can be curatively treated through surgical resection with appropriate lymphadenectomy. Nevertheless, a subset of patients experience postoperative recurrence.^3)^ While adjuvant chemotherapy can reduce the risk of recurrence, its efficacy in Stage II (UICC TNM, 8th edition) remains inconclusive. Depending on the site of recurrence, secondary surgical resection may offer a favorable prognosis,^4)^ however, outcomes remain poor with non-surgical treatments.^5)^ Consequently, the management of unresectable disease continues to present a significant clinical challenge.

Approximately 20% of patients present with metastatic disease at diagnosis, and 25%–35% develop metastasis during the course of their illness.^6)^ Ocular metastases are exceedingly rare. Among choroidal metastases, the most common primary sites are the lung (47%) and breast (21%), while gastrointestinal primaries account for only 4%.^7)^

Herein, we report a rare case of choroidal metastasis from colorectal cancer in which long-term survival was achieved through combined local therapies. We also review the clinical characteristics of choroidal metastases originating from colorectal cancer based on a literature review.

## CASE PRESENTATION

**Fig. 1:**
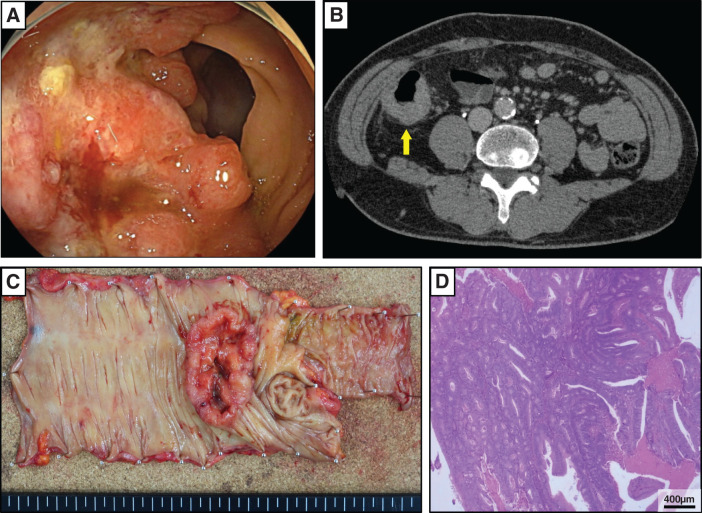
Preoperative examination and postoperative specimens, pathological examination. A type 2 tumor (well to moderately differentiated adenocarcinoma) was identified in the ascending colon (**A**), and CT examination revealed circumferential wall thickening but no feathering around the tumor (**B**, yellow arrow). No enlarged lymph nodes were detected in the surrounding area. Laparoscopic right hemicolectomy with D3 lymph node dissection was performed (R0 resection, pT3N0M0, pStage IIa) (**C**). Pathological examination revealed well to moderately differentiated adenocarcinoma (tub1 > tub2, INFb, Ly0, V0) (**D**).

A 67-year-old man was diagnosed with ascending colon cancer (T3N0M0, Stage IIA [UICC TNM 8th edition]) (**Fig. 1A** and **1B**) following a screening examination. He underwent laparoscopic right hemicolectomy with CME, achieving R0 resection (**Fig. 1C**). Histopathological examination revealed a well to moderately differentiated adenocarcinoma (tub1 > tub2, INFb), with no evidence of lymphovascular invasion (Ly0, V0) (**Fig. 1D**). Molecular profiling showed wild-type *RAS* and *BRAF*, and microsatellite stability, indicating a low risk of recurrence. After discussion with the patient, adjuvant chemotherapy with capecitabine (3600 mg/day) plus oxaliplatin (130 mg/m^2^) was administered for four cycles, followed by surveillance.

**Fig. 2:**
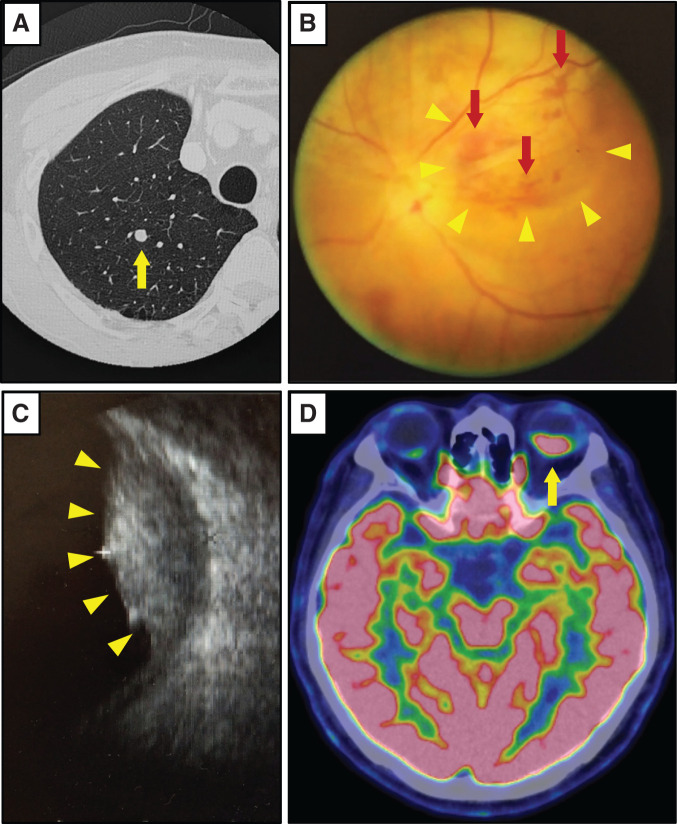
Images when diagnosing choroidal metastasis in the left eye. A 2 mm nodule was detected in the upper lobe of the right lung (**A**, yellow arrow). Fundus examination revealed a greyish-white tumor shadow (yellow arrowheads) with partial surface bleeding (red arrows) on the outer side of the left optic nerve head (**B**), and intraocular ultrasound examination detected an 11 mm thick tumor in the choroidal layer (**C**, yellow arrowheads). PET-CT examination revealed abnormal accumulation in the left choroid (**D**, yellow arrow).

At 1 year and 10 months postoperatively, a metastatic lesion was detected in the right upper lobe of the lung, for which partial resection was performed. At 2 years and 5 months postoperatively, two additional pulmonary metastases were identified (**Fig. 2A**). Around the same time, the patient began experiencing visual impairment, visual field defects, and mild ocular pain in the left eye, prompting further evaluation.

Ophthalmologic examination revealed a grayish-white mass with superficial retinal hemorrhage located lateral to the optic disc on fundoscopic inspection (**Fig. 2B**). Ocular ultrasonography demonstrated an 11-mm thick choroidal mass (**Fig. 2C**). PET/CT showed abnormal uptake in the left choroid (**Fig. 2D**), leading to a diagnosis of choroidal metastasis. Systemic chemotherapy with capecitabine (3000 mg/day) plus bevacizumab (7.5 mg/kg) was initiated, and irinotecan (200 mg/m^2^) was added starting in the second month of treatment.

**Fig. 3:**
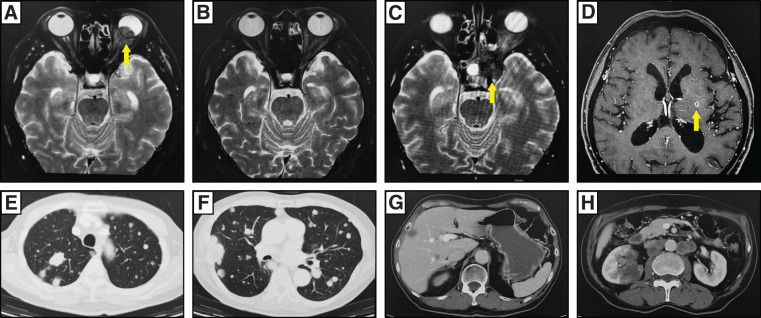
Images during treatment. The treatment progress of metastatic lesions in the head was compared using MRI. At 19 months after ocular metastasis, the metastatic lesion increased in size (**A**, yellow arrow). At 22 months, the metastatic lesion had almost disappeared (**B**). At 28 months, the metastasis had infiltrated the orbit, skull, and paranasal sinuses (**C**, yellow arrow). At 29 months, metastasis was detected in the left basal ganglia (**D**, yellow arrow). Multiple systemic metastases were identified by CT scan. Multiple metastatic lung tumors were found throughout both the lungs (**E** and **F**), with liver metastasis (**G**), right kidney metastasis, and para-aortic lymph node metastasis also present (**H**).

After 5 months of therapy, the choroidal tumor had shrunk to 2 mm, and ocular pain had nearly completely resolved. However, irinotecan was discontinued after 11 months due to treatment-related nausea (Grade 2, CTCAE v5.0). By 15 months, progressive tumor enlargement was observed, accompanied by worsening ocular pain and fundus hemorrhage. By 19 months, invasion beyond the globe—including into the optic nerve—was evident (**Fig. 3A**), and local control was deemed unachievable. As a result, localized radiotherapy to the left eye was administered (50 Gy/5 Fr).

By 22 months, the ocular pain had nearly resolved, and the tumor demonstrated a tendency to regress (**Fig. 3B**). However, radiation-induced complications, including ophthalmoplegia and ptosis, developed. At 28 months, tumor progression and invasion into the orbit, skull, and paranasal sinuses were noted (**Fig. 3C**), prompting additional radiotherapy to the skull base (35 Gy/5 Fr). By 29 months, marked regression of the orbital and skull lesions was achieved; however, a new metastatic lesion was identified in the left basal ganglia (**Fig. 3D**), which was treated with stereotactic radiotherapy (24 Gy/1 Fr).

**Fig. 4:**
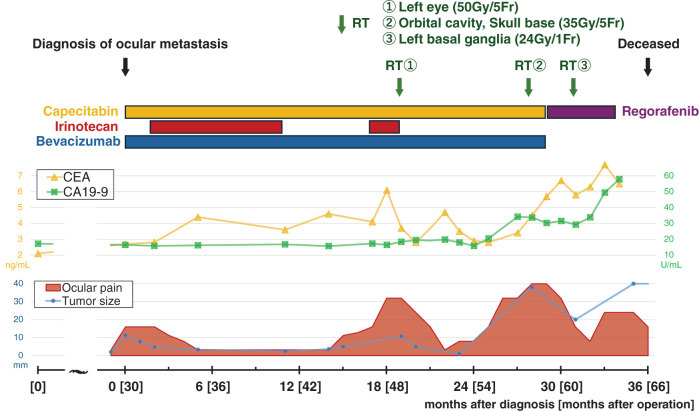
Clinical course table after diagnosis of choroidal metastasis. Upper section: Clinical events, treatment details; Middle section: Tumor marker trends; Lower section: Trends in the size of the ocular tumor and ocular pain (relative indicators).

Systemic therapy was switched to regorafenib (80 mg/day), but disease progression continued, with enlargement of pulmonary metastases. By 33 months, widespread disease was evident, including multiple lung metastases, liver metastases, para-aortic lymphadenopathy, and right renal metastases (**Fig. 3E**–**3H**). Systemic disease was no longer controllable, and the patient was transitioned to palliative care. He passed away 5 years and 6 months after the initial surgery and 3 years after the diagnosis of choroidal metastasis (**Fig. 4**).

## DISCUSSION

Most cases of colorectal cancer with choroidal metastasis are already in a state of multi-organ metastasis at the time of diagnosis, and the 3-year survival rate for colorectal cancer with choroidal metastasis is approximately 14%.^8)^ Treatment options for such cases include systemic chemotherapy, which remains the standard of care for metastatic colorectal cancer, as well as local ocular therapies such as radiotherapy, intravitreal bevacizumab injection, and enucleation.^9)^ While these local therapies do not necessarily restore vision, they play an important role in slowing visual deterioration and alleviating symptoms such as ocular pain, thereby improving quality of life.

To better understand the clinical features of choroidal metastasis from colorectal adenocarcinoma, we conducted a literature search using PubMed and Google Scholar covering the period from 2000 to 2025. We identified 19 case reports^6,10–27)^ and 1 case series,^28)^ yielding a total of 21 cases. Including our own, we analyzed 22 patients in total (**Table 1**).

**Table 1:** Cases on choroidal metastasis from colorectal primary cancer

No.	Year	Study	Age	Sex	Primary site	Symptoms at presentation	Period until diagnosis (month)	Histology type	Other metastatic sites	Treatment	Period since diagnosis (month)	Clinical outcome
1	2000	Ward^10)^	52	F	Colon	Vision loss	0	NS	Peritoneum	None	1	Dead
2	2004	Linares^11)^	47	M	Rectum	Vision loss, Visual disturbances	0	Por	Lung, liver, bone	CRT (FOLFOX)	9	Dead
3	2005	Apte^12)^	39	M	Colon	Visual disturbances	3	Por	Lung, liver	CT (NS) + Operation + RT	NS	NS
4	2006	Hisham^13)^	32	F	Rectum	Eye pain	10	Por	Brest, bone	RT	2	Dead
5	2008	Kuo^14)^	65	F	Colon	Vision loss	20	NS	Brain	BV Injection	6	Alive
6	2010	Lin^15)^	43	M	Colon	Vision loss	96	NS	Bone	BV Injection	4	Dead
7	2010	Neale^16)^	43	M	Rectum	Visual disturbances	18	Mod	Lung, peritoneum, brain	None	0	Dead
8	2014	Tei^17)^	60	M	Rectum	Vision loss	30	Well	Lung	RT + CT (CapeOX + Bev)	27	Alive
9	2015	Huo^18)^	51	F	Colon	Blurred vision	27	NS	Lung, bone	RT	3	Dead
10	2015	Khawaja^6)^	60	F	Rectum	Vision loss, Visual disturbances	48	Well	Brain, lung, bone, adrenal gland	RT + CT (FOLFOX + Bev)	32	Dead
11	2015	Maudgil^28)^	57	F	Colon	Vision loss	18	Mod	None	BV Injection	NS	NS
12	2015	Maudgil^28)^	80	M	Colon	Vision loss	6	NS	Liver	BV Injection	NS	Dead
13	2016	Boss^19)^	68	F	Rectum	Blurred vision	96	NS	Lung, brain	BV Injection	NS	NS
14	2016	Ha^20)^	78	F	Colon	Visual disturbances	18	NS	Brain, Lung, skin	CT (Cap)	8	Dead
15	2016	Nookala^21)^	56	M	Colon	Vision loss, Eye pain	24	Well	Lung, liver	RT	21	Alive
16	2017	Walker^22)^	54	M	Colon	Vision loss	16	Mod	Lung	RT + CT (FOLFIRI + Bev)	24	Dead
17	2020	Kegel^25)^	72	F	Rectum	Vision loss	16	Por	Lung, liver, bone	RT	20	Dead
18	2020	Neto^23)^	70	M	Colon	Vision loss	8	NS	Lung, liver	CT (FOLFILINO + Bev) + PDT	1	Alive
19	2020	Sánchez^24)^	64	M	Colon	Vision loss, Eye pain	36	Mod	Lung	Ope	6	Dead
20	2021	Amisha^26)^	44	M	Rectum	Vision loss	0	NS	Lung, liver	CT (FOLFOX) + RT	0	Alive
21	2024	Demir^27)^	74	M	Rectum	Vision loss	21	Mod	Lung, brain	RT	2	Dead
22	2025	Current case	67	M	Colon	Vision loss	30	Well	Lung	CT (CAPIRI + BV, REG) + RT	36	Dead

BV, bevacizumab; CT, chemotherapy; F, female; M, male; Mod, moderately differentiated; NS, not specified; Por, poorly differentiated; REG, regorafenib; RT, radiotherapy; Well, well differentiated

Among these cases, 13 were male (59.1%), with a mean age of 58.5 years (range: 32-80), and 13 had the colon as the primary tumor site (59.1%). Most patients had multiple distant metastases at the time of choroidal involvement. In our case as well, pulmonary metastases had already been documented. The most common metastatic sites were the lungs (16 cases, 72.7%), liver (8 cases, 36.4%), brain (6 cases, 27.3%), and bone (6 cases, 27.3%), suggesting hematogenous spread as the predominant route of metastasis.

Although systemic chemotherapy was administered in 9 cases (40.9%), local ocular treatments—including radiotherapy, intravitreal bevacizumab, or surgical excision—were utilized in 19 cases (86.4%). This underscores the importance of local control, as ocular metastasis can severely impair patients’ quality of life through elevated intraocular pressure and pain. In our case, repeated radiation therapy with treatment modification allowed for sustained local control of the intraocular tumor, despite multiple episodes of tumor progression.

The average survival following the diagnosis of intraocular metastasis was 10.4 months, with a median of 6 months (range: 0–32 months). Although the prognosis is generally poor due to the presence of expensive metastatic disease at diagnosis, several cases of long-term survival have been reported. Notably, our case represents the longest reported survival following the diagnosis of choroidal metastasis from colorectal cancer to date and serves as an important clinical example of the potential for long-term local management of ocular lesions.

## CONCLUSIONS

This case report highlights the clinical characteristics of choroidal metastasis from colorectal cancer, a condition increasingly reported in the literature. It offers important insights into diagnosis and treatment decision-making. Notably, this case demonstrates that long-term survival with preserved quality of life can be achieved through a combination of systemic therapy and local treatment, particularly radiotherapy targeting the ocular lesion.

In the context of long-term management of metastatic colorectal cancer, clinicians should remain aware of the potential for intraocular metastasis. Treatment strategies should not rely solely on systemic therapy but should also incorporate local modalities—such as radiotherapy—to achieve local control and symptom relief.

## ACKNOWLEDGMENTS

We would like to thank Editage (www.editage.jp) for English language editing.

## DECLARATIONS

### Funding

No funding was received.

### Authors’ contributions

KR and TK conceptualized the case report, and KR drafted the manuscript.

TK, HT, YZ, and EO were involved in the treatment and follow-up.

TK, EO, and TY critically revised the manuscript and provided valuable feedback.

TY supervised the project and approved the final version for submission.

All authors have read and approved the final version of the manuscript.

### Availability of data and materials

The data generated during the present study are available from the corresponding author upon reasonable request.

### Ethics approval and consent to participate

This case report describes an individualized treatment based on the patient’s condition and clinical evidence. Therefore, it is classified as an exempt study and does not require formal ethical approval. Informed consent was obtained, and the study adhered to ethical standards, including respect for patient privacy.

### Consent for publication

Informed consent for the publication of individual data and accompanying images was obtained from the patient.

### Competing interests

The authors declare that they have no competing interests.
